# Recent Progress of Atomic Layer Technology in Spintronics: Mechanism, Materials and Prospects

**DOI:** 10.3390/nano12040661

**Published:** 2022-02-16

**Authors:** Yuanlu Tsai, Zhiteng Li, Shaojie Hu

**Affiliations:** Center for Spintronics and Quantum Systems, State Key Laboratory for Mechanical Behavior of Materials, Xi’an Jiaotong University, Xi’an 710049, China; andycai@stu.xjtu.edu.cn (Y.T.); qq619755720@stu.xjtu.edu.cn (Z.L.)

**Keywords:** spintronics, atomic layer technology, atomic layer etching, atomic layer deposition, self-limiting, reaction mechanism, atomic scale

## Abstract

The atomic layer technique is generating a lot of excitement and study due to its profound physics and enormous potential in device fabrication. This article reviews current developments in atomic layer technology for spintronics, including atomic layer deposition (ALD) and atomic layer etching (ALE). To begin, we introduce the main atomic layer deposition techniques. Then, in a brief review, we discuss ALE technology for insulators, semiconductors, metals, and newly created two-dimensional van der Waals materials. Additionally, we compare the critical factors learned from ALD to constructing ALE technology. Finally, we discuss the future prospects and challenges of atomic layer technology in the field of spinronics.

## 1. Introduction

With the introduction of FinFET transistor technology, which successfully solved the problem of transistor leakage, the entire semiconductor industry was able to break beyond the technological barrier of around 20 nm technology in the twentieth century. GAA (gate-all-around) architecture has recently pushed the limit of semiconductors to 2 nm, which is the smallest size currently attainable in terms of physical dimensions. To a significant extent, the advancement of this technology has aided in the development of advanced semiconductor processes, which has brought the technology ever closer to the physical limitations of its capabilities. As a result, storage and computing systems based on the complementary metal–oxide–semiconductor (CMOS) design will be unable to keep up with the demands of today’s and tomorrow’s information explosion. The efforts to overcome these challenges by the creation of a new paradigm for architecture, devices, and materials, have been fraught with obstacles.

A ray of hope for the development of new logic devices, new memory devices, and new forms of integrated devices is provided by spintronics, which will fall beyond the limits of the CMOS framework but will nonetheless be useful. There are many different types of devices, including spin FETs, all spin logic, domain wall logic, spin–wave logic, magnetic random access memory (MRAM), and nano-spin oscillators, to name a few examples. Emergent materials, namely, magnetic materials, strong spin–orbital coupling metals, and topological materials, are required to realize such devices. Magnetic materials, in particular, are required. MRAM is the spintronics product that has had the most commercial success in this industry. Magnetron sputtering for atomic-level controlled film growth is the most widely used technology in the industrial fabrication of magnetic random access memory. In practice, however, the old dry etching approach is still employed for the etching process, which cannot achieve the atomic layer level etching required for the procedure. The use of atomic layer etching technology will be required in order to scale the size of the unified cell for MRAM. At the moment, atomic layer etching methods are primarily focused on semiconductors and certain oxide insulators, with just a small amount of study being done on simple magnetic metal substances and alloys. Our introducing the technology in a systematic manner will allow the reader to gain a thorough understanding of atomic layer etching technology and how to apply it to new materials. In atomic layer etching, the challenge lies in the fact that we must use different chemical reagents for different materials. In order to overcome this challenge, researchers must design specific chemical reagents for the etching of unique metal materials and alloys, which is also one of the most difficult aspects of atomic layer etching technology.

Our review aspires to provide a complete overview of atomic layer technology while also illuminating the various principles that underpin it. Following this quick introduction, we go through the mechanisms of ALD and the ALE approach. In Section 2, we provide an overview of the most recent ALD technology. It is discussed in Section 3 how ALE technology can be used for several types of materials’ systems. These materials include insulators, semiconductors, metals, and the recently developed two-dimensional van der Waals materials. In addition, we compare the major distinctions that we can learn from the ALD in order to develop ALE technology in the future. Finally, we discuss the potential and problems for the field of atomic layer technology in the foreseeable future.

## 2. Atomic Layer Deposition Technique

The technology of atomic layer film deposition can be broadly classified into two groups. The first is physical vapor deposition (PVD), in which a thin film is formed on the surface by a physical process using a material source. It encompasses a variety of techniques, including molecular beam epitaxy (MBE), magnetron sputtering (MS), Joule heating evaporation and electron-beam heating evaporation, pulsed laser deposition (PLD), etc. [1], The second is chemical vapor deposition (CVD), in which a precursor is deposited onto the surface and performs a chemical reaction to generate a thin film.

### 2.1. Molecular Beam Epitaxy (MBE)

**Figure 1:**
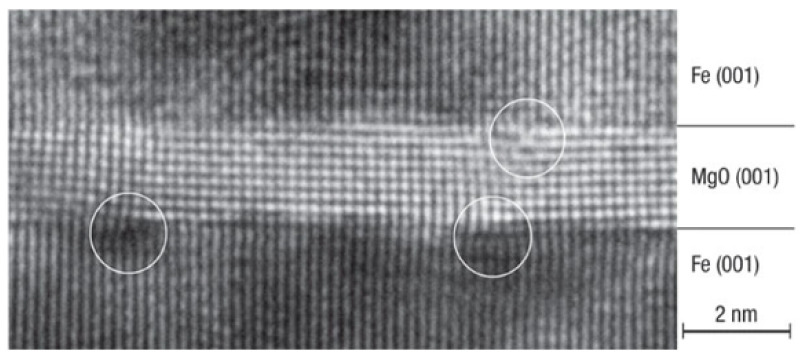
TEM images of a single-crystal MTJ with the Fe (001)/ MgO (001) (1.8 nm)/Fe (001) structure fabricated by MBE. Reprint with permission form Ref. [5]. Copyright 2004 Springer Nature.

Molecular beam epitaxy is a kind of ultra-high vacuum thin film growth technique which uses directed neutral thermal atoms and molecular beams to hit a heated substrate and interact with the substrate surface to grow single crystal thin films [2,3]. It can be traced back to the late 1960s, Arthur et al. used to grow III-V semiconductors such as gallium arsenide (GaAs) [4]. Following that, MBE ushered in a new era of atomic layer growth of high-quality single crystal materials, bringing in the age of nanotechnology. This approach is also effective for the growing of heterojunction thin films. High spin polarized ferromagnetic signal crystal materials, such as the half metal, Heusleralloy, topological insulator, and a few magnetic tunneling junctions (MTJ), have been developed as a result of the development of spintronics. The MTJ, the key structure of MRAM, dominates the future performance of devices. A single-crystal MTJ with the Fe/MgO/Fe structure fabricated by MBE shown the giant room temperature tunneling magnetoresistance (TMR) effect [5]. The TEM picture demonstrates that the interface junction is extremely well regulated at the atomic layer level shown in Figure 1.

In 2D magnetic materials research, which is a branch of spintronics, the MBE technique is making rapid advancements at the present. Firstly, the intrinsic ferromagnetism of 2D magnets was only detected in low-temperature conditions, which was a significant step forward. In 2018, O’Hara et al. observed ferromagnetism in ${\mathrm{MnSe}}_{x}$ films grown by MBE at room temperature [6,7]. A representative metal 2D magnet is represented by the monolayerr ${\mathrm{VSe}}_{2}$, which exhibits charge density wave and magnetic phenomena at the same time. At the same time, the monolayerr ${\mathrm{VSe}}_{2}$ with charge density wave and magnetic phenomena is a representative metal 2D magnet. According to the results of their research, Wong et al. created a monolayer of ${\mathrm{VSe}}_{2}$ on highly oriented pyrolytic graphite (HOPG) using MBE to seek for novel low-dimensional quantum phenomena [8,9]. MBE was used to build a room temperature ferromagnetic monolayer ${\mathrm{Cr}}_{3}{\mathrm{Te}}_{4}$ in 2021 by Chua et al. at a lower substrate temperature [10]. In conclusion, these findings demonstrate that MBE is still a highly successful and important method for generating thin films at the atomic layer level. However, the ultrahigh vacuum growing conditions, high purity source material, and sluggish growth rate of this technology are the primary obstacles to its practical deployment in the industrial setting [3,9].

### 2.2. Magnetron Sputtering

Magnetron sputtering is a deposition technique that uses a gaseous plasma that is created and confined to a space holding the material to be deposited. High-energy ions in the plasma erode the target’s surface, and the released atoms travel through the vacuum environment and deposit on a substrate to grow a thin film. The growing rate of the thin film can be changed by applying a bias at the substrate position to control the bombardment and energy of the incident material. Due to the high deposition rate and scalability, it is now the most extensive technology for depositing metal and compound thin films and has many industrial applications. However, the composition of the deposited film may be different from the target (source) material [11,12,13].

The sputtering technology can be traced back to the mid-19th century, when Grove first discovered the sputtering was a ‘dirt effect’ [14]. Due to the low deposition rate in the plasma, etc, there was a huge obstacle in the fabrication of nanomaterials at the beginning. It was not until the 1970s that the introduction of an external magnetic field was well resolved, and the deposition rate was greatly increased [1,15]. In recent years, due to the exploration and development of non-metallic and non-conductive thin films, magnetron sputtering has also developed new concepts, including dual magnetron sputtering, high-power pulsed magnetron sputtering, DC magnetron sputtering and radio frequency magnetron sputtering, etc. [16,17,18,19,20,21,22,23]. In the current era of sustainable development, magnetron sputtering technology is considered to be an important means of fabricating high-performance electrode materials for electrochemical energy storage (EES) devices. Besides, the textiles deposited by MS is also a way to give special properties such as antibacterial and UV resistance to textiles used in people’s lives [24,25].

**Figure 2:**
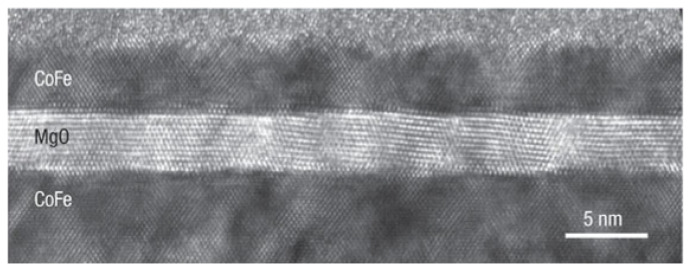
TEM images of a single-crystal MTJ with a highly oriented (100) MgO tunnel barrier fabricated by MS. High-resolution images along the [110] zone axes showing atomically resolved lattice planes with (100) planes perpendicular to the growth direction. Reprint with permission from Ref. [39]. Copyright 2004 Springer Nature.

Magnetron sputtering has had a significant impact on the application of hard wear-resistant coatings, coatings with special optical or electrical properties, and has become the preferred process for deposition of important industrial thin-film coatings. At the same time, it also has an important impact in the field of electrocatalysis [11,26]. The materials of magnetron sputtering usually include: pure metals, alloys, chalcogenides, carbides, nitrides, non-metal, etc. [25,27,28,29,30,31,32]. Besides in the field of spintronics, magnetron sputtering is an important method for the fabrication of magnetic tunnel junctions [33,34,35]. The early research of MTJ started from Julliere in 1975. He discovered the TMR effect in the Fe/Ge/Co-based magnetic tunnel junction structure. However, due to the small TMR value and can only be achieved at low temperature, the application of MTJ is limited [36]. After Miyazaki et al. discovered a large TMR effect at room temperature in 1995, the study of MTJ was widely discussed [37,38]. The atomic layer controlled CoFe/MgO/CoFe MTJ shown in Figure 2 was developed by S.S Parkin until 2004 using MS method. Atomically resolved lattice planes with (100) planes perpendicular to the growth direction can be seen in high-resolution images taken along the [110] zone axes of the [110] zone [39]. With the introduction of ferromagnetic materials such as CoFeB in the later period, the fabrication of MTJ has developed rapidly. In 2005, Djayaprawira et al. achieved the fabrication of MTJ based on ${\mathrm{Co}}_{60}$${\mathrm{Fe}}_{20}$$B_{20}$/MgO/${\mathrm{Co}}_{60}$${\mathrm{Fe}}_{20}$$B_{20}$ at room temperature, and the TMR value reached 230% [40]. Nowadays, different magnetron sputtering processes have been widely used in the fabrication of magnetic tunnel junctions. By adjusting the magnetic field distribution of the cathode target and optimizing the target source, the problem of low utilization of magnetron sputtering targets has also been improved [41].

As an important method for depositing metal and non-metal thin films. Magnetron sputtering can deposit high-density thin films at low temperature and high rate under environmentally friendly conditions. It will also be developed in more fields in the future.

### 2.3. Chemical Vapor Deposition (CVD)

The term chemical vapor deposition was first mentioned in the book by Powell et al. in 1966 [42]. It was invented in the late 1970s under the name of atomic layer epitaxy [43] and applied to thin-film electroluminescent (TFEL). ALD is considered a type of CVD. The term ALD is used throughout this section to refer to the CVD-based atomic layer deposition process. With the development of microelectronics, the shrinking of integrated circuits, and the increase in aspect ratio, ALD is now widely used due to its self-limiting and layer-by-layer deposition advantages [44].

**Figure 3:**
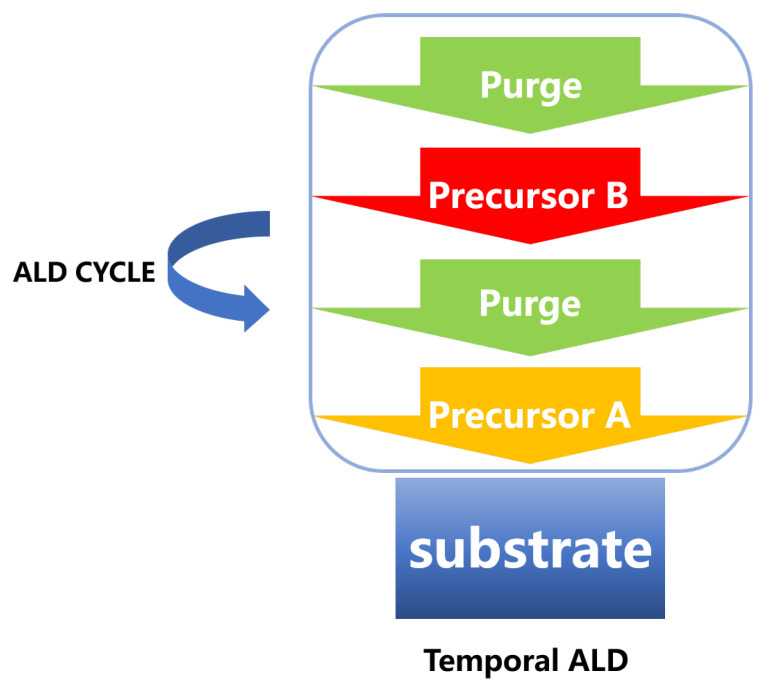
Schematic illustrations of one typical ALD cycle. First, the precursor A is introduced to the chamber to be chemically adsorbed on the substrate, and then the by-products and excess precursors are removed by the inert gas. Last, the precursor B is introduced to react with the adsorbed molecules to form the monolayer of the desired material before cleaning again by the inert gas.

Generally, the ALD process can be divided into two steps as shown in Figure 3. In the first step, a chemical adsorption or reaction occurred with the surface of the substrate by introducing the first precursor, and then the inert gas is introduced to remove the excess precursors and by-products before entering the second step. In the second step, the second precursor is introduced to react with the first precursor on the substrate to form a thin film. Furthermore, after the reaction reaches the expectation, an inert gas is introduced to remove the by-products and excess precursors produced in the second step, thereby achieving a reaction cycle. Since the film produced in each cycle is a monoatomic layer in an ideal state, a self-limiting effect can be achieved, which facilitates the control of the deposition thickness. Now that ALD has gone through many years of research, a series of materials such as oxide materials, two-dimensional materials, semiconductor materials, and metal materials can be used to prepare thin films on specific substrates. In the research process of these films, they exhibited many required properties. For example, the earliest article published by Kim et al. 1995 used Silicon (IV) chloride (${\mathrm{SiCl}}_{4}$) and $H_{2}O$ for ALD reaction to deposit a very thin Silicon dioxide (${\mathrm{SiO}}_{2}$) film on porous Vycor glass [45], due to its choice of hydrogen permeability, it has many applications in the water gas shift reaction and other catalysis processes involving hydrogen [46].

In the field of metal ALD, Daub et al. used nickelocene and cobaltocene as the first precursor and $O_{3}$ as the second precursor to prepare nickel (Ni) and cobalt (Co) nanotubes in the aluminum oxide film in 2007 [47]. They found that after using the $O_{3}$ as the second precursor to deposit, the size of the reduced crystal grain structure is very small, making the thickness control more feasible. The prepared nanotubes have attracted wide attention in the fields of sensor devices and magnetic imaging due to their properties [48]. In the field of two-dimensional material ALD, Zhang et al. developed a remote plasma-enhanced atomic layer deposition (PEALD) in 2014 to grow graphene on copper foil using benzene as a carbon source at a low temperature of $400{\;}^{\circ }$C [49]. The prepared graphene has important applications in the fields of optoelectronic devices due to its excellent physical properties [50,51,52]. In the field of oxide material ALD, Cheng et al. used ${\mathrm{TiCl}}_{4}$ and $H_{2}O$ as precursors to prepare ${\mathrm{TiO}}_{2}$ thin-film ALD process in 2008 [53]. The ${\mathrm{TiO}}_{2}$ film produced has a high application value in water splitting photocatalysis and other fields [54].

Since the successful fabrication of graphene [55], 2D materials have attracted great attention in recent years due to their unique dimensions and related properties. Compared with 3D materials, 2D materials show an ultra-high ratio of surface area to volume. At the same time, the quantum confinement, strong spin–orbit coupling and quantum spin Hall effects caused by their unique dimensions produces many interesting properties. The ultra-thin structure and excellent electronic properties of 2D materials also make it an ideal component of nanoelectronics in the future [56,57,58,59]. In order to realize the wide application of 2D materials, how to manufacture high-quality 2D materials on a large scale has become the research direction of many scholars. Generally, 2D materials are prepared by methods such as mechanical exfoliation, chemical exfoliation, and vapor growth [60,61,62,63,64]. The mechanical exfoliation method can prepare high-quality 2D materials, but it is obviously not suitable for large-scale production and relies heavily on the experience of researchers. Compared with top-down exfoliation, bottom-up vapor deposition has the advantages of mass production of 2D materials and high-quality control [65]. Table 1 summarizes some major ALD process parameters and applications.

To date, the application of ALD in spintronics has gradually become a hot spot [83,84], especially in the coupling between ferromagnetic (FM) thin films and topological insulators (TI) [85,86], because it could provide conformal growth on top of even granular TIs. Longo et al. [87] successfully grow cobalt films with controllable thickness on ${\mathrm{Sb}}_{2}{\mathrm{Te}}_{3}$ substrates using the bis (1,4-ditert-butyl-1,3-diazadienyl) cobalt and tert-butylamine (${\mathrm{tBuNH}}_{2}$) at $180{\;}^{\circ }$C, and studied the magnetic properties of Co/${\mathrm{Sb}}_{2}{\mathrm{Te}}_{3}$ heterostructures [88].

**Figure 4:**
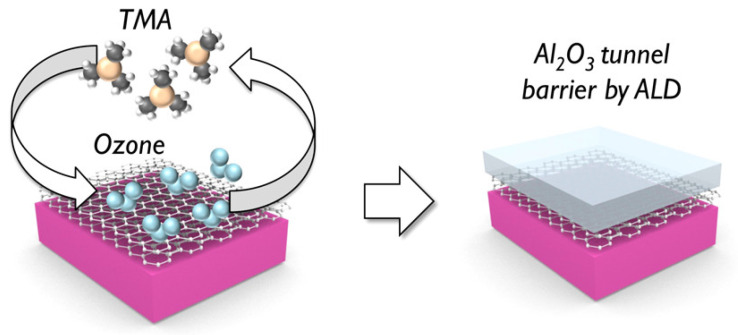
Schematic illustrations of the ozone-based atomic layer deposition process on graphene-coated Ni electrodes. Ni electrodes exposed to air. Through the ALD cycle, by pulsing of ozone and trimethylaluminum (TMA), the electron-transparent ${\mathrm{Al}}_{2}O_{3}$ tunnel barrier is finally formed. Reprint from Ref. [83].

**Figure 5:**
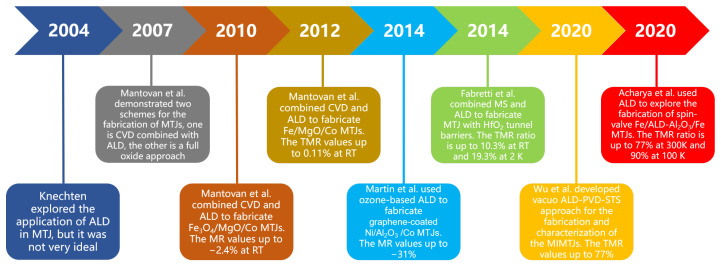
Timeline for the development in the use of ALD for MTJ [83,92,93,94,95,96,98,100].

ALD is undoubtedly a key technology for growing high-quality, uniform and ultra-thin films. However, for the field of MTJ in spintronics, the advantages of ALD are still not widely used. The fabrication of MTJ mostly relies on relatively complex physical deposition, although there have been some pioneering attempts [89,90,91]. A key reason is that in the process of ALD, the metallic spin sources will be oxidized, resulting in a decline in spintronic performance. Therefore, the ALD process based on water or ozone has not been well applied in the fabrication of MTJ. Knechten first introduced the ALD-${\mathrm{Al}}_{2}O_{3}$ barriers MTJs in 2004. As the extremely low pinhole density and homogeneity are not fulfilled in these structures, the bottom electrode probably may been damaged. At that time ALD for MTJ fabricated seems not promising [92]. Mantovan et al. started the research on the fabrication of MTJ using ALD earlier. They demonstrated two schemes for the fabrication of MTJs, one is CVD combined with ALD, the other is a full oxide approach and they successfully fabricated ${\mathrm{Fe}}_{3}O_{4}$/MgO/Co MTJs and Fe/MgO/Co MTJs through the combination of CVD and ALD [93,94,95]. In 2014, Martin et al. realized the preparation of graphene-coated $\mathrm{Ni}/{\mathrm{Al}}_{2}O_{3}/\mathrm{Co}$ magnetic tunnel junction based on sub-nanometer, ozone-based ALD and the tunneling characterization shows that it has a high-quality electron transport barrier [83]. Figure 4 shows the ozone-based ALD-grown tunnel barriers. Fabretti et al. compared the TMR ratio, bias voltage, and temperature dependence of MTJs prepared by ALD with ${\mathrm{HfO}}_{2}$ as barrier material and MgO-based and Al-based MTJs [96]. Wilt et al. explored the use of ALD to deposit 1–6 Å thick ${\mathrm{Al}}_{2}O_{3}$ tunnel barriers on Fe films with ultra-thin Al wetting layers [97]. Wu et al. developed vacuo atomic layer deposition-physical vapor deposition-scanning tunneling spectroscopy (ALD-PVD-STS) approach for the fabrication and characterization of the metal-insulator-metal tunnel junctions (MIMTJs) and they used it to investigate Fe/${\mathrm{Al}}_{2}O_{3}$/Fe magnetic tunnel junctions with very small ${\mathrm{Al}}_{2}O_{3}$ thickness to 0.2 nm [98]. Recently, Acharya et al. realized the fabrication of $\mathrm{Fe}/\mathrm{ALD}\text{-}{\mathrm{Al}}_{2}O_{3}/\mathrm{Fe}$ MTJs with 0.55 nm $\mathrm{ALD}\text{-}{\mathrm{Al}}_{2}O_{3}$ TBs by self-designed situ ALD [99,100]. ALD provides an important solution for the fabrication of ultrathin (sub-nm 1 nm), leak-free and defect-free TBs. Figure 5 shows the timeline of the development in the use of ALD for MTJ. In the future, with the further development of MRAM, the application of ALD in this area will also receive more attention.

Compared with the PVD process, the CVD process has the advantages of the conformally deposited film, a wide variety of deposited materials, and the high purity, but it also has the disadvantages that different precursors have different special properties, the CVD process relies on high-temperature reaction conditions and others [101]. Up to now, CVD has formed a basic theoretical framework in many material synthesis fields. However, a lot of research is still needed to simulate the most suitable synthesis conditions for various materials, and to discuss special phenomena in the synthesis process [102,103].

## 3. Mechanism of Atomic Layer Etching

**Figure 6:**
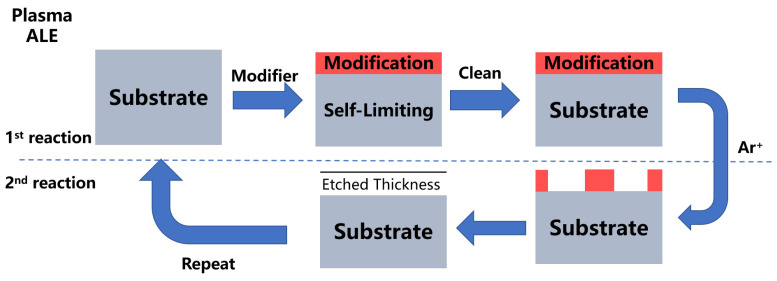
Illustrations of a typical cycle of plasma ALE in the form of a diagram. In the 1st reaction, the substrate is affected by the modified gas and subsequently cleaned by the inert gas. The high-energy particles are utilized in the 2nd reaction to etch the modification, and the cycle is completed following the inert gas cleaning steps.

**Figure 7:**
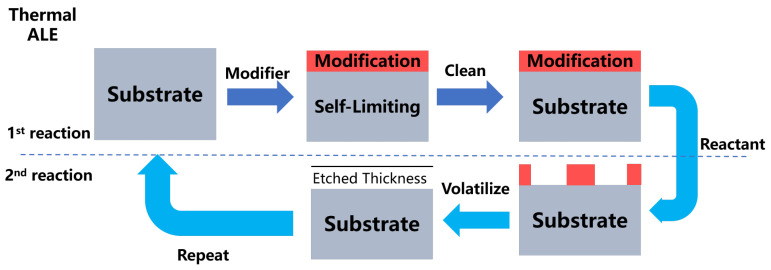
Illustrations of a typical thermal ALE cycle in the form of a schematic diagram. In the 1st reaction, the substrate is affected by the modified gas and subsequently cleaned by the inert gas. The 2nd reaction employs an active reactive gas to react with the modification, after which the volatile products are released, and the cycle is completed after the inert gas cleaning steps.

It is possible to precisely etch materials layer-by-layer using the ALE process, which has the potential to be used to etch materials without causing damage or contamination. It is a cyclic etch process in which chemical adsorption and physical desorption are carried out in a consecutive manner. ALE is always thought of as the reversal of the ALD procedure. There is a link between it and Yoder’s 1988 U.S. patent on the subject. A single atomic layer of a synthetic diamond film is removed off the surface of the film using this process [104]. As it was considered to be a high-power semiconductor material at the time, GaAs was extensively investigated for use in the fabrication of integrated circuit substrates, infrared detectors, gamma photon detectors, body-effect devices, and other applications [105]. Due to this, the approach developed by Maki et al. to self-limit etch the atomic level surface layer of GaAs is a further study of the ALE method [106]. Sakaue et al. employed fluorine or chlorine to adsorb on the Si surface in 1991, and they were the first to demonstrate self-limited etching of the Si film surface layer by argon (Ar) ion bombardment at room temperature in 1992 [107]. This type of operation is referred to as plasma atomic layer etching (plasma ALE). Meanwhile, Steven M. George published the first report on a novel ALE method dubbed thermal atomic layer etching in 2015. He was the first to describe the technique [108]. To summarize, ALE may be categorized into two types: anisotropic plasma ALE shown in Figure 6, and isotropic thermal ALE shown in Figure 7. The ALE process, like the ALD process, consists of two steps. In the ideal conditions of the first step, the modified gas modifies the surface, resulting in the creation of a self-limiting single-layer reaction film. To prevent contamination during the reaction step, inert gas will be channeled into the reaction chamber to clean it and remove any unreacted modified gas. The second step is where the primary distinctions between plasma ALE and thermal ALE occur. The plasma ALE process does this by removing the reactive layer and etching the surface with high-energy particles. The thermal ALE achieves the purpose of removing the reactive coating by reacting on the surface with an active reactive gas. Finally, the reaction chamber is cleaned once more in the second step to complete an ALE cycle.

Each ALE technique has distinct advantages. In the second stage of the plasma ALE, the ion energy is critical for the actual etching process. Whereas when the ion energy is too low, the modified layer is not completely removed, when it is too high, the etching depth exceeds the modified layer, increasing the roughness on the modified layer [109]. As a result, the notion of the ALE window has been developed. Etch per cycle (EPC) is independent of ion energy in some specific energy range, and the best solution is achieved at this point. Therefore, the regulation of ion energy is extremely crucial in order to ensure that ALE is kept within its own self-limitation limits. However, for the majority of the thermal ALE reactions that have been described, the EPC is significantly affected by temperature, and the ALE window is difficult to observe due to its short width [110,111,112]. Another essential element is the reactive gas, as different material systems require varied amounts of surface modification gas. The selection of a surface modification gas is quite challenging, as the modified material’s binding energy requires quite different energy for bombardment particles. As a result, it is necessary to conduct independent research on a variety of material systems in order to ascertain the similarities and differences in their operating parameters, and then to discover certain universal rules. Following that, we take a systematic look at insulators, semiconductors, metals, and two-dimensional materials.

## 4. Materials for ALE

### 4.1. Insulators

#### 4.1.1. Oxide Materials

As it well known, oxides are the most prevalent and are the most frequently utilized insulator material in micro- and nanoscale processes. The capacitance between wires and heat generation are frequently encountered during integrated circuit research. To minimize them, oxide films with a high dielectric constant are frequently employed, including ${\mathrm{SiO}}_{2}$ and aluminium oxide (${\mathrm{Al}}_{2}O_{3}$), zinc oxide (ZnO), titanium dioxide (${\mathrm{TiO}}_{2}$), and many others. This section summarizes the most frequently used oxide film etching techniques. The study on ALE on oxide films began with a US patent issued in 1994 by Jeng et al., which demonstrated the use of ALE to etch ${\mathrm{SiO}}_{2}$ films [113].

**Figure 8:**
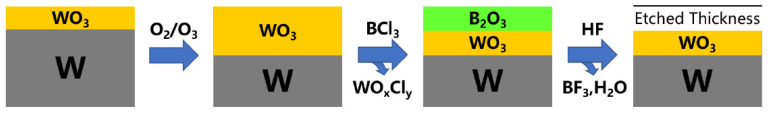
A schematic of the oxidation–conversion–fluorination method for W and ${\mathrm{WO}}_{3}$. First, the W is oxidated by the $O_{2}$/$O_{3}$. Second, the ${\mathrm{WO}}_{3}$ is conversed to $B_{2}O_{3}$ by ${\mathrm{BCl}}_{3}$ to form ${\mathrm{WO}}_{x}{\mathrm{Cl}}_{y}$ products. Last, the $B_{2}O_{3}$ is fluorinated by HF to form the volatile ${\mathrm{BF}}_{3}$ and $H_{2}O$.

At the moment, the conversion-fluorination and fluorination-ligand-exchange processes are the most frequently utilized methods. While conversion fluorination is commonly used for oxide materials such as ZnO, ${\mathrm{TiO}}_{2}$, ${\mathrm{SiO}}_{2}$, and tungsten trioxide (${\mathrm{WO}}_{3}$), fluorination-ligand exchange is typically used for oxide materials such as ${\mathrm{Al}}_{2}O_{3}$, hafnium (IV) oxide (${\mathrm{HfO}}_{2}$), and zirconium dioxide (${\mathrm{ZrO}}_{2}$). Table 2 summarizes the ALE process parameters for the major oxide materials. The reactants in the producing and removing modifier steps, respectively, are surface adsorption and surface removal. The oxidation–conversion–fluorination method was derived from this process in recent years [114]. The tungsten (W) plays a significant role in the field of chip manufacture. The following section briefly describes the etching principle of W and how this process works. As illustrated in Figure 8, the W was first oxidized by $O_{2}$/$O_{3}$.

(1) $$W+3\;O_{3}\;\left(g\right)\to \;WO_{3}+3\;O_{2}\;\left(g\right),$$

(2) $$W+\frac{3}{2}O_{2}\;\left(g\right)\to \;WO_{3},$$

Then it is transformed by BCl${}_{3}$:

(3) $$WO_{3}+2\;BCl_{3}\;\left(g\right)\to B_{2}O_{3}+WCl_{6}\;\left(g\right),$$

(4) $$WO_{3}+\frac{4}{3}\;BCl_{3}\;\left(g\right)\to \frac{2}{3}\;B_{2}O_{3}+WOCl_{4}\;\left(g\right),$$

(5) $$WO_{3}+\frac{2}{3}\;BCl_{3}\;\left(g\right)\to \frac{1}{3}\;B_{2}O_{3}+WO_{2}Cl_{2}\;\left(g\right),$$

and then fluorination by HF:

(6) $$B_{2}O_{3}+6\;HF\left(g\right)\to 2\;BF_{3}\;\left(g\right)+3\;H_{2}O\;\left(g\right),$$

According to the results of the calculations, the activation energies of hydrogen fluoride (HF) and ${\mathrm{WO}}_{3}$, and W, boron trichloride (${\mathrm{BCl}}_{3}$) and W at 200 °C are all greater than zero, indicating that they will not react independently during the etching process [114]. This finding is extremely beneficial for today’s metal oxide research since it allows us to better understand different etching mechanisms.

#### 4.1.2. Nitride Materials

Along with the research on oxide materials mentioned previously, scientists have also taken an interest in nitride materials. The majority of nitride films are used as critical components in semiconductor devices, such as TiN as a diffusion barrier layer, AlN as a complementary metal-oxide-semiconductor gate electrode, ${\mathrm{Si}}_{3}N_{4}$ as an isolator, mask, diffusion barrier layer, dielectric and protective layer in micro-electromechanical system (MEMS) devices. The first ALE studies on nitride films were conducted in 1999 by Matsuura et al. [124]. It was discovered that the interaction of hydrogen atoms with the ${\mathrm{Si}}_{3}N_{4}$ surface could remove nitrogen atoms. The interaction was achievable by bombarding the modified surface alternately with Ar and hydrogen ions.

**Figure 9:**
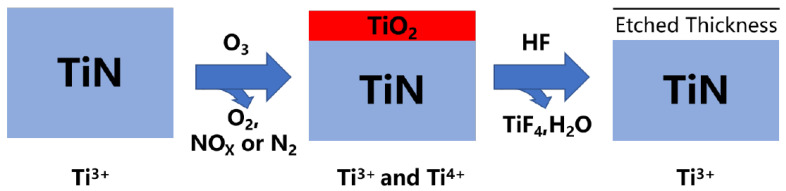
A schematic of etching reaction of TiN. First, the TiN is oxidated by the $O_{3}$ to the ${\mathrm{TiO}}_{2}$. Last, the ${\mathrm{TiO}}_{2}$ is fluorinated by HF to form the volatile ${\mathrm{TiF}}_{4}$ and $H_{2}O$.

Nowadays, HF is used in the etching process of nitride layers, but it serves a different purpose. As an illustration, the etching process of TiN can be characterized as the oxidation-fluorination method shown in Figure 9. The first step is the oxidation of surface TiN using $O_{3}$.

(7) $$TiN+3\;O_{3}\to TiO_{2}+NO+3\;O_{2}$$

The high oxidizability $O_{3}$ enables it to spontaneously change trivalent Ti to tetravalent Ti. Additionally, $O_{3}$ can oxidize TiN to generate the self-limiting diffusion barrier ${\mathrm{TiO}}_{2}$. The second procedure is to fluorinate ${\mathrm{TiO}}_{2}$ with HF.

(8) $$TiO_{2}+4\;HF\to TiF_{4}+2\;H_{2}O$$

At room temperature, the reaction has the most favorable thermodynamic conditions, and the products ${\mathrm{TiF}}_{4}$ and $H_{2}O$ are highly flammable. Then repeat one or two stages to accomplish atomic layer etching [110]. In addition, the fluorination ligand exchange and oxidation fluorination ligand exchange techniques are frequently employed for nitride film etching. The process parameters for several common nitride films are listed in Table 3. Despite the fact that there is a dearth of study on nitride materials such as ${\mathrm{Si}}_{3}N_{4}$, TiN, and AlN. It is difficult to etch them using the traditional etching procedure, “conversion-fluoridation.” However, they had been etched using the “oxidation–conversion–fluorination” technique. Further investigation of the etching mechanism may enable the development of significantly more ALE techniques on nitride materials.

### 4.2. Semiconductors

**Figure 10:**
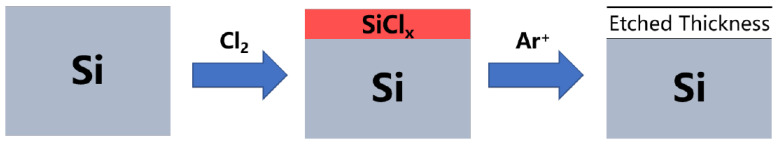
A schematic of the etching reaction of Si. First, the Si is modified by ${\mathrm{Cl}}_{2}$ to form the ${\mathrm{SiCl}}_{x}$. Last, the ${\mathrm{SiCl}}_{x}$ surface is removed by the high-energy Ar ion.

In light of the importance of etching in the fabrication of semiconductor chips, the etching technology of semiconductor thin films is a critically essential study area in the field of semiconductor manufacturing. The etching of Si is considered to be the first scientific investigation. This has resulted from the in-depth study of Si that has taken place in recent years. As a result of these discoveries in germanium, carbon, GaN, and other semiconductor materials, the scientific community has achieved consistent progress in these areas. At this point in time, they have progressed to a relatively advanced stage of development in the plasma ALE technology [127]. Significant advancements have been made in the study of atomic layer etching of silicon since its inception in 1990 [128]. As of today’s date, the most advanced approach for the removal of silicon atoms from its atomic layer is to use argon ions after the silicon has been changed with chlorine gas. However, it is uncertain if ${\mathrm{SiCl}}_{4}$ or ${\mathrm{SiCl}}_{2}$ is produced and the schematic of Si ALE is shown in Figure 10 [129]. When each ion participates in the reaction independently, the neutral synergistic impact of the ions can be effectively controlled, and self-limitation to a higher extent can be realized, allowing for more effective control of the reaction process.

In recent years, researchers have concentrated their efforts on the high-power semiconductors silicon carbide (SiC) and gallium nitride (GaN). Research on GaN and other III-V binary semiconductor materials began in 1999 with the publication of a US patent by Bozada et al., which set the framework for later investigation. The surface of GaN is oxidized, and then the surface is removed using a dilute acid bath [130]. Ohba et al. have published the ALE etching method for GaN, which is now more widely used. Finally, the cyclic procedure of chemical adsorption through ${\mathrm{Cl}}_{2}$ plasma and then etching through Ar ions can achieve an etching rate of 0.4 nm/cycle after several cycles [131]. According to a recent study, Lee et al. employed ${\mathrm{SF}}_{6}$ and $H_{2}$ to perform atomic layer etching of SiC. They were successful in attaining an etching rate of 0.4 Å/cycle at an RF power of 300 W [132]. Based on the preceding report, we summarize the etching parameters for germanium, carbon, GaN, and the other semiconductors indicated in Table 4 before moving on to the next section. In recent years, high-power transistors have grown increasingly significant as the semiconductor industry has developed at a breakneck pace. High-power semiconductors can be difficult to process, especially when they are large. Looking ahead, ALE is very compatible with the realization of device fabrication and interconnection under lower nano nodes As a result, research into semiconductor ALE will continue to play a vital role in the future of chip’s fabrication.

### 4.3. Metals

Metallic films offer excellent physical features, including high electrical and thermal conductivity, and are therefore commonly utilized in metal gate electrodes and connection layers. However, many heavy metals can generate the nonvolatile etching by-products after etching process because of chemically inert properties. All of these factors enhance the difficulty of metal etching. If ALE’s study overcomes these obstacles, it will have a huge impact on how metals are used in integrated circuits. Currently, the majority of ALE approaches for metal films are also two-step processes [141]. The first stage is to change the metal film into the aforementioned metal oxide, nitride, or chloride. The second step is then based on self-limiting reactions to form organic and easily-removable ligands. Meanwhile, the second phase may occasionally involve plasma therapy. As a result, we can choose the most optimal ALE procedure via an appropriate solution. Simultaneously, research on the metal ALE and metal ALD cycle procedures has found that the processes are relatively comparable. Utilizing the metal’s thermally driven ALD to forecast its thermally driven ALE process may be an efficient strategy for extending the ALE process. We explain the ALE etching technique for metal thin films in the next sections, focusing on two aspects of single-element metals and alloys, and summarize the pertinent parameters in Table 5.

#### 4.3.1. Signal Element Metals

In 2000, Kuo et al. published the first report on a two-step dry etching technique for copper (Cu). To produce copper etching, the Cu surface was chlorinated first and then immersed in dilute hydrochloric acid to dissolve the ${\mathrm{CuCl}}_{x}$ product [156]. Recent years, the thermochemical ALE of W has been significant improved. W was initially etched mostly via oxidation conversion fluorination. In 2019, Kim et al. suggested a method for fluorination oxidation, in which a layer of ${\mathrm{WF}}_{y}$ was produced at room temperature using ${\mathrm{NF}}_{3}$ plasma, and then the volatile ${\mathrm{WO}}_{x}F_{y}$ was made during the removal process by oxygen plasma reacting with the surface layer. Although the oxygen plasma would react with W, the exposure period of W was kept to less than or equal to 30 s/cycle by maintaining the oxygen plasma’s voltage at 30 V [150].

Additionally, Fe, Co, Ni and other magnetic metal materials have garnered considerable attention in recent years. Umezaki et al. submitted a paper on IEEE NANO in 2016 describing a thermal dry etching procedure for nickel etching using Hhfac (1,1,5,5,5-hexafluoro-2,4-pentanedione). The entire process begins with the oxidation of Ni by oxygen to generate nickel oxide, which is subsequently reacts with Hhfac to achieve etching [157]. Furthermore, in 2020, Basher et al. investigated the formation and desorption of nickel hexafluoroacetylacetonate $\mathrm{Ni}{\left(\mathrm{hfac}\right)}_{2}$ on a nickel oxide surface using atomic layer etching operations [155].

In the surface modification step, the Ni film may be exposed to the oxygen plasma and oxidized to a thin nickel oxide (NiO) layer.

(9) Ni+O→NiO

In the surface removal step, the NiO layer is exposed to gaseous hexafluoroacetylacetone (hfacH) and then form the volatile nickel hexafluoroacetylacetonate $\mathrm{Ni}{\left(\mathrm{hfac}\right)}_{2}$ and $H_{2}O$

(10) $$NiO+2\;hfacH\to Ni{\left(hfac\right)}_{2}+H_{2}O$$

**Figure 11:**
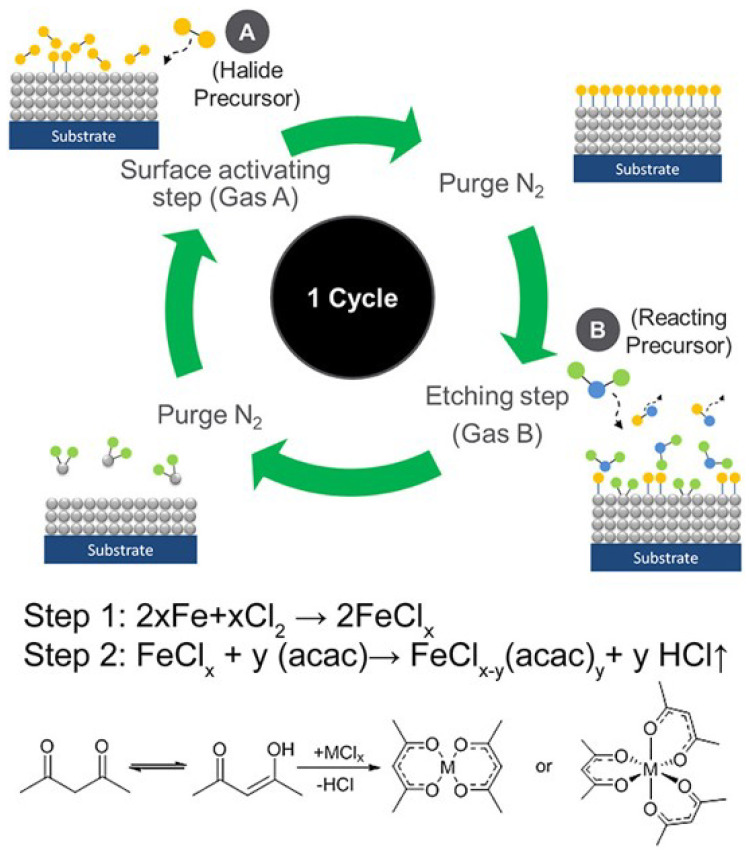
Schematic illustrations of one typical Fe ALD cycle. In the first step, Fe is chlorinated with chlorine to form ${\mathrm{FeCl}}_{x}$. In the second step, the ${\mathrm{FeCl}}_{x}$ is reacted with acetylacetone (acac) to form volatile metal complexes. The gases A and B are a halogen precursor ${\mathrm{Cl}}_{2}$ and an organic precursor acetylacetone (acac). Reprint with permission from Ref. [146]. Copyright 2018 American Vacuum Society.

In 2018, Lin et al. published the etching of Fe by alternately reacting chlorine and acetylacetone (acac) to form volatile metal complexes at low temperatures [146]. The whole Fe ALE process is shown in Figure 11.

In the experiment, chlorine gas was used instead of oxygen to modify the material, because the ${\mathrm{MO}}_{x}$ residue of oxygen etching may reduce the magnetization of magnetic tunnel junction (MTJ). Thus, another process will be needed to removed it by sputtering [158]. In 2019, Konh et al. reported the etching mechanism of a Co film in continuous exposure to chlorine and diketone (either 1,1,1,5,5,5-hexafluoro-2,4-pentanedione (hexafluoroacetylacetone, hfacH) or 2,4-pentanedione (acetylacetone, acacH)). They found that the best conditions for Co’s ALE process require the presence of surface chlorine and surface oxygen. The hfach is a more effective etchant than acacH and the product of the simultaneous etching process, contains ${\mathrm{Co}}^{3+}$ with multiple ligands [147]. Metal ALE research is still in its early stages, and there is still a considerable lack in understanding.

#### 4.3.2. Alloys

In practical applications such as semiconductor devices, single-element metals often cannot meet actual needs due to interference from various factors such as the service environment. The alloy needs to be investigated further in order to match the demand and get higher performance. However, due to the fact that the alloy contains distinct element compositions, we must use separate etching techniques. There are significant problems in the etching process, such as surface modification and etching self-restriction, among other things. There have been very few studies conducted on the etching of alloys using ALE. When it comes to alloy etching concepts, we believe that we should look for suitable modifiers that will allow us to meet the self-limiting needs of etching with varied compositions. Furthermore, by carefully managing the temperature, ion energy, and other factors, the variation in etching rate between different components may be kept within an acceptable range of possibilities.

In 2020, the etching of SiGe alloy through thermal ALE was reported. Li et al. introduced the self-limiting oxidation of ${\mathrm{Si}}_{0.72}{\mathrm{Ge}}_{0.28}$ through oxygen plasma, and then the self-limiting cyclic etching through ${\mathrm{CF}}_{4}$/$C_{4}F_{8}$ alternately can reach 2.3 Å/cycle [159]. In 2021, a new ALE etching process for SiGe alloy was reported by Abdulagatov et al. [160]. They introduced the self-limiting oxidation of ${\mathrm{Si}}_{0.15}{\mathrm{Ge}}_{0.85}$ through $O_{2}$/$O_{3}$ at $290{\;}^{\circ }$C through the oxidation–conversion–etching mechanism. Then, through HF and TMA conversion-etching, it can reach 0.57 Å/cycle and 0.42 Å/cycle, respectively. According to its atomic force microscope (AFM) images, the SiGe film’s surface is not roughened.

**Figure 12:**
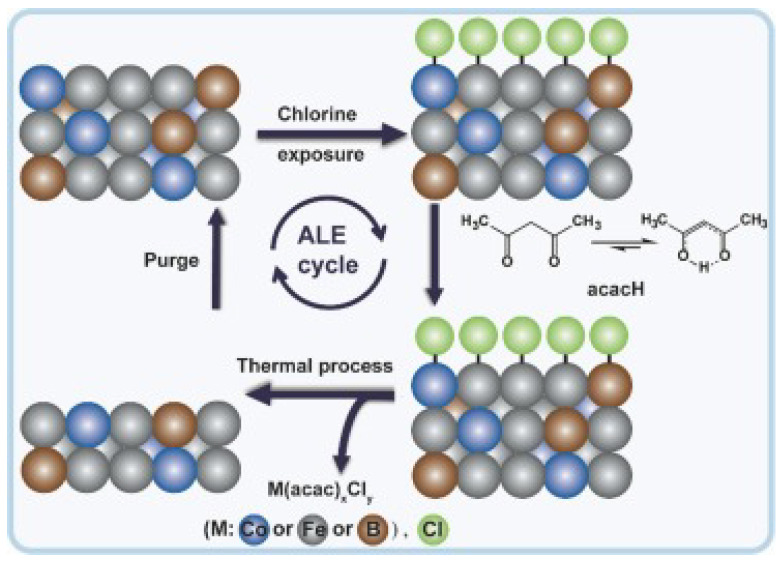
The proposed general approach and viable reactions during the CoFeB alloy atomic layer etching cycle using sequential exposure of chlorine and acacH. Reprint with permission from Ref. [164]. Copyright 2022 Elsevier.

We have mentioned before we can use MBE and MS to fabricate the MTJ for MRAM. Fe, Co, Ni, and their alloys are typically required as raw materials in the fabrication of MTJ. CoFeB alloy is a common material in the ferromagnetic free layer and fixed layer of MTJ and has been shown to be capable of interacting with the MgO tunneling barrier, which is a characteristic of this group of materials [161]. The etching of CoFeB alloy is a time-consuming and challenging process. Previously, ion beam milling (IBE) was a popular method of etching metals due to its high efficiency. While this method would deposit sputtering material on the sidewall of the tunnel junction feature, it would result in electrical shorts and poor etching selectivity during the etching operation [162]. It is anticipated that when the ALE technique is used to this end, there will be a significant improvement. For the etching of the CoFeB film, Altieri et al. published a paper in 2019 reporting an etching rate of 1.8 nm/cycle, achieved by using an alternating cycle of oxygen plasma with a −100 V substrate bias and formic acid vapor [163]. Most recently, Konh et al. employed a continuous dosage of chlorine and 2,4-pentanedione (acetylacetone, acacH) to etch a CoFeB alloy film, and achieved an etching rate of 0.15 nm/cycle using the thermal dry etching method. It has also been established that MgO can have a protective effect on CoFeB alloy, allowing it to be employed in the manufacturing of MTJ [164]. The whole CoFeB alloy ALE process is shown in Figure 12.

In summary, there is still considerable space for advancement in the research of alloys utilizing the ALE approach. ALE etching of metal thin films is typically preceded by oxidation or chlorination, followed by removal using organic ligands such as formate, carboxylate, acetylacetonate (acac), or hexa-fluoroacetylacetonate (hfac), among others. An alloy is a collection of metals, and the ALE process for an alloy can be compared with that for a single-element metal.

### 4.4. Two-Dimensional (2D) van der Waals Materials

In 2004, graphene that can exist stably at room temperature was first discovered through the mechanical exfoliation method [55]. The 2D van der Waals material has dimensions at the nanometer level in the three-dimensional space (that is, close to the thickness of an atomic layer). As the thickness of a certain dimension has reached the atomic level, the quantum size effect appears, and there are many interesting physical and chemical characteristics. These characteristics are very important for the research of next-generation electronic and optoelectronic devices. However, there are still just a few studies on ALE on 2D van der Waals materials. In the following section, we provide a quick overview of the ALE etching procedure for graphene. As well as other gathered alloys and 2D materials, the ALE etching process parameters for these materials are listed in Table 6.

Graphene, as a classic 2D van der Waals material, has been extensively studied, and the research in the field of ALE etching is growing vigorously. A widely-recognized ALE cyclic etching process for graphene was reported in 2017 [165]. It uses controlled low-energy (0–20 eV) oxygen ions ($O^{2+}$/$O^{+}$) for chemical modification, and then uses low-energy (11.2 eV) argon ion beams to perform the chemical modification. The modified layer is removed, and graphene with a controllable number of layers can be obtained. By comparing the atomic force microscope images and Raman spectroscopy before and after the ALE etching, they think that there is almost no obvious damage on the surface of the graphene after the etching is completed.

The current state of 2D materials research is extremely active. When it comes to producing integrated devices based on 2D materials in the future, ALE technology will be a critical component of the overall process. Consequently, much in the way of labor and material resources must be dedicated to the creation and research of the ALE process for 2D materials.

## 5. The Connection between ALD and ALE

At the present, the ALE process is still in the early stages of development. In comparison to the more established ALD technique, there is still a great deal of space for development of ALE. ALE is the reverse process of ALD. The existing ALD research can provide us with some valuable insights and practical experience for developing ALE technique. Since ALE is widely regarded as the etching counterpart of ALD, scientists have been trying to find the relationship between the two to realize that through one study, multiple results of different processes can be obtained [169]. We also carry out some conclusions about the relationship between them. First of all, both are atomic-level manipulation processes. Both can be divided into two half-reactions in the process steps, and there is a sweeping step between each half-reaction. We can also observe that during the reaction process both are self-limiting. Last but not least they both have a variety of surface reactions during the reaction process. The various cycle types of ALD are suitable for ALE. ALE still has the problem of directionality. We can use ALD to deposit masks to protect sidewalls. Therefore, we can use ALD to deposit complex patterns of masks to protect areas that we do not want to be etched. Furthermore, because of the ion synergy [170], the effect will be improved.

**Figure 13:**
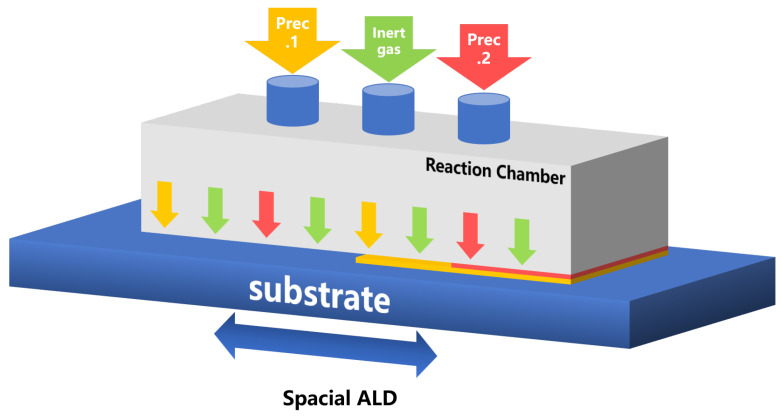
A schematic of spatial ALD. Differently from temporal ALD, the substrate can swing between different areas.

In addition, there is the concept of spatial ALD that has not been widely mentioned [171]. We think it can also be applied ALE, generating spatial ALE, although the first patent involving atmospheric pressure SALD can be traced back to 1983 and was proposed together with ALD. The first report on the application of the SALD method was published in 2004 [172], and industrial commercialization has been achieved [173,174,175,176]. In temporal ALD, the precursors are sequentially exposed to the substrate through short pulses while maintaining physical separation through an intermediate cleaning step. However, in the SALD shown in Figure 13, the substrate swings between different areas through the control system to achieve spatial separation. Through analysis and research on fluid dynamics and modeling, the reactor was designed and the optimal deposition conditions were evaluated, and the research proved that as long as high precursor partial pressure and a high molar flow rate can be achieved, atmospheric pressure SALD can be used to achieve high yield coated porous substrates [177]. Compared with conventional ALD, the speed of SALD can be increased by up to two orders of magnitude. Furthermore, the possibility of performing SALD under atmospheric pressure (AP-SALD) or even in the open air (no deposition chamber is used at all) makes it cheaper and easier to scale up, because complex and expensive vacuum processing is not required. Therefore, the ALE field could also be relevant.

## 6. Prospects and Challenges

ALE is now trailing behind ALD in terms of technology advancement. These two methods are vital for the fabrication of chips and sensors at the same time, but the materials involved are quite complex, particularly those associated with composite materials such as alloys and heterojunctions. Metal alloys and two-dimensional materials are significant among them. They contain many different elements though, so the etching procedures for them are not as simple as for basic substances. More individuals need to get involved and continue to explore new techniques in order to solve the new difficulties that have arisen as a result of the rapid growth of integrated circuits.

## Figures and Tables

**Table 1:** ALD for some common materials.

Materials Deposited by ALD	Reactants of the ALD Process	Substrate	Application	Reference
${\mathrm{SiO}}_{2}$	${\mathrm{SiCl}}_{4}/H_{2}O$	Silica glass	Gas separation	[45]
${\mathrm{SiO}}_{2}$	${\mathrm{SiCl}}_{4}/H_{2}O$	AAO	Microfluidics	[66]
Ni, Co	${\mathrm{NiCp}}_{2}/H_{2}O$, ${\mathrm{CoCp}}_{2}/O_{3}$	AAO	Sensing	[47]
Al	TMA	MOFs	Catalysis, separation	[67]
Pt	${\mathrm{MeCpPtMe}}_{3}$	MOF MIL-101	Catalysis/Direct TEM imaging	[68,69]
${\mathrm{Al}}_{2}O_{3}$	$\mathrm{TMA}/H_{2}O$	Natural collagen membranes	Improved mechanical properties	[70]
${\mathrm{TiO}}_{2}$	$\mathrm{TTIP}/H_{2}O$	Natural collagen membranes	Improved mechanical properties	[70]
ZnO	$\mathrm{DEZ}/H_{2}O$	Natural collagen membranes	Improved mechanical properties	[70]
CoS	$\mathrm{Co}{\left(\mathrm{amd}\right)}_{2}/H_{2}S$	MOFs	Catalysis	[71]
Graphene	$C_{6}H_{6}/H_{2}$ plasma	Cu	High electron mobility, flexibility high optical transparency superior mechanical strength	[49]
BN	${\mathrm{BBr}}_{3}/{\mathrm{NH}}_{3}$	Silica	Wide band gap	[72]
BN	${\mathrm{BCl}}_{3}/{\mathrm{NH}}_{3}$	${\mathrm{ZrO}}_{2}$	high stability	[73,74]
BN	$\mathrm{TEB}/{\mathrm{NH}}_{3}$	Sapphire or Si	excellent thermal conductivity	[75]
${\mathrm{MoS}}_{2}$	${\mathrm{MoCl}}_{5}/H_{2}S$	${\mathrm{SiO}}_{2}/\mathrm{Si}$	Electronic and optical devices	[76]
${\mathrm{MoS}}_{2}$	${\mathrm{MoCl}}_{5}/H_{2}S$	${\mathrm{SiO}}_{2}/\mathrm{Si}$ and quartz	energy conversion, energy storage and solid-state lubrication	[77]
${\mathrm{WS}}_{2}$	$W{\left(\mathrm{CO}\right)}_{6}/H_{2}S$	${\mathrm{SiO}}_{2}/\mathrm{Si}$	Solid lubricant	[78]
${\mathrm{TiO}}_{2}$	${\mathrm{TiCl}}_{4}/H_{2}O$	AAO	Biosensing	[79]
${\mathrm{Al}}_{2}O_{3}$	$\mathrm{TMA}/H_{2}O$	Porous silver/ Carbonate matrix	${\mathrm{CO}}_{2}\;\mathrm{capture}$	[80]
${\mathrm{Al}}_{2}O_{3}$	$\mathrm{TMA}/H_{2}O$	MF ${\mathrm{ZrO}}_{2}\;\mathrm{membranes}$	Water filtration	[81]
${\mathrm{ZrO}}_{2}$	$\mathrm{TDMAZr}/H_{2}O$	Porous silver/ carbonate matrix	${\mathrm{CO}}_{2}\;\mathrm{capture}$	[82]

**Table 2:** Oxide materials.

Materials	Etch Type	Surface Adsorption	Surface Removal	EPC (Å/cycle)	Reference
${\mathrm{SiO}}_{2}$	Thermal ALE	HF	TMA	0.31	[115]
	Plasma ALE	${\mathrm{CHF}}_{3}$	${\mathrm{Ar}}^{+}$ (50 eV)	5 (Synergy = 80%)	[116]
${\mathrm{WO}}_{3}$	Thermal ALE	$O_{2}/O_{3}$	${\mathrm{BCl}}_{3}\left(40\;\mathrm{mTorr}\right)\to$ HF (60 mTorr)	4.18	[114]
${\mathrm{HfO}}_{2}\left(\mathrm{crystalline}\right)$	Thermal ALE	HF	${\mathrm{TiCl}}_{4}$	0.02	[117]
${\mathrm{HfO}}_{2}\left(\mathrm{amorphous}\right)$	Thermal ALE			0.36	[117]
${\mathrm{Al}}_{2}O_{3}$	Thermal ALE	HF	$\mathrm{Sn}{\left(\mathrm{acac}\right)}_{2}$	0.61	[118]
	Thermal ALE	${\mathrm{NbF}}_{5}$	${\mathrm{CCl}}_{4}$	1.4	[119]
	Thermal ALE	HF	TMA	0.14/0.75	[120]
${\mathrm{HfO}}_{2}$	Thermal ALE	HF	$\mathrm{Sn}{\left(\mathrm{acac}\right)}_{2}$	0.117	[111]
	Thermal ALE	HF	$\mathrm{Al}{\left({\mathrm{CH}}_{3}\right)}_{2}\mathrm{Cl}$	0.77	[121]
${\mathrm{ZrO}}_{2}$	Thermal ALE	HF	${\mathrm{SiCl}}_{4}/\mathrm{Sn}{\left(\mathrm{acac}\right)}_{2}$	0.14	[121]
	Thermal ALE	HF	$\mathrm{Al}{\left({\mathrm{CH}}_{3}\right)}_{2}\mathrm{Cl}$	0.117	[121]
ZnO	Thermal ALE	HF	TMA	2.19	[122]
${\mathrm{TiO}}_{2}$	Thermal ALE	${\mathrm{WF}}_{6}$	${\mathrm{BCl}}_{3}$	0.6∼0.7	[112]
${\mathrm{WO}}_{3}$	Thermal ALE	${\mathrm{BCl}}_{3}$	HF	4.19	[114]
${\mathrm{Ga}}_{2}O_{3}$	Thermal ALE	HF	${\mathrm{BCl}}_{3}$	0.59–1.35	[123]
	Thermal ALE	HF	$\mathrm{AlCl}{\left({\mathrm{CH}}_{3}\right)}_{2}$	1.2	[123]
	Thermal ALE	HF	$\mathrm{Al}{\left({\mathrm{CH}}_{3}\right)}_{3}$	0.82	[123]
	Thermal ALE	HF	${\mathrm{TiCl}}_{4}$	0.85	[123]
	Thermal ALE	HF	$\mathrm{Ga}{\left(N{\left({\mathrm{CH}}_{3}\right)}_{2}\right)}_{3}$	0.23	[123]

**Table 3:** Nitride materials.

Materials	Etch Type	Surface Adsorption	Surface Removal	EPC (Å/cycle)	Reference
TiN	Thermal ALE	$O_{3}$	HF	0.2	[110]
	Thermal ALE	$H_{2}O_{2}$	HF	0.15	[110]
AIN	Thermal ALE	HF	$\mathrm{Sn}{\left(\mathrm{acac}\right)}_{2}$	0.36	[125]
	Thermal ALE	HF	$\mathrm{Sn}{\left(\mathrm{acac}\right)}_{2}/$$H_{2}$ plasma	1.96	[125]
${\mathrm{Si}}_{3}N_{4}$	Thermal ALE	$O_{2}\left(250\;\mathrm{Torr}\right)$	HF(0.65 Torr) + TMA(1.2 Torr)	0.25	[126]
	Thermal ALE	$O_{2}\left(450\;\mathrm{Torr}\right)$	HF(0.65 Torr) + TMA(1.2 Torr)	0.25	[126]
	Thermal ALE	$O_{3}\left(250\;\mathrm{Torr}\right)$	HF(0.65 Torr) + TMA(1.2 Torr)	0.47	[126]
	Thermal ALE	$O_{3}\left(250\;\mathrm{Torr}\right)$	HF(0.40 Torr) + TMA(1.2 Torr)	0.38	[126]
	Thermal ALE	$O_{3}\left(250\;\mathrm{Torr}\right)$	HF(0.65 Torr) + TMA(0.6 Torr)	0.38	[126]
	Thermal ALE	$O_{3}\left(250\;\mathrm{Torr}\right)$	HF(0.81 Torr) + TMA(1.2 Torr)	0.5	[126]

**Table 4:** Semiconductors.

Materials	Etch Type	Surface Adsorption	Surface Removal	EPC (Å/cycle)	Reference
Si	Plasma ALE	${\mathrm{Cl}}_{2}$	${\mathrm{Ar}}^{+}\left(50\;\mathrm{eV}\right)$	7 (Synergy = 90%)	[116]
	Thermal ALE	$O_{2}\to$ $F_{2}$	TMA	0.4	[133]
Ge	Plasma ALE	${\mathrm{Cl}}_{2}$	${\mathrm{Ar}}^{+}\left(25\;\mathrm{eV}\right)$	8 (Synergy = 66%)	[116]
C	Plasma ALE	$O_{2}$	${\mathrm{Ar}}^{+}$ (50 eV)	3.1 (Synergy = 97%)	[116]
Ga	Plasma ALE	${\mathrm{Cl}}_{2}$	Ar^+^ (70 eV)	3.3 (Synergy = 91%)	[116]
InP	Plasma ALE	${\mathrm{Cl}}_{2}$	Ne		[134]
GaAs	Plasma ALE	${\mathrm{Cl}}_{2}$	Ar		[135]
GaN	Plasma ALE	${\mathrm{Cl}}_{2}$	Ar		[136]
	Thermal ALE	${\mathrm{XeF}}_{2}$	${\mathrm{BCl}}_{3}$		[137]
	Plasma ALE	${\mathrm{Cl}}_{2}$	${\mathrm{Ar}}^{+}\left(70\;\mathrm{eV}\right)$	3.3	[116]
InGaAs	Plasma ALE	${\mathrm{Cl}}_{2}$	Ar		[138]
	Thermal ALE	HF-pyridine	$\mathrm{AlCl}{\left({\mathrm{CH}}_{3}\right)}_{2}$		[139]
AlGaN	Plasma ALE	${\mathrm{Cl}}_{2}$	Ar		[140]

**Table 5:** Metals.

Materials	Etch Type	Surface Adsorption	Surface Removal	EPC (Å/cycle)	Reference
Cr	Thermal ALE	$O_{2}$	${\mathrm{Cl}}_{2}$	1.1	[142]
	Plasma ALE	${\mathrm{Cl}}_{2}$	Ar	2	[142]
Pd	Thermal ALE	VUV and $O_{2}$	Formic acid	2.81	[143]
	Plasma-Thermal ALE	$O_{2}$		12	[144]
Ta	Thermal ALE	$O_{2}$	Ethanol		[145]
Pt	Plasma-Thermal ALE	$O_{2}$	Formic acid	5	[144]
		$O_{2}$	Ethanol		[145]
Fe	Thermal ALE	${\mathrm{Cl}}_{2}$	acac	50	[146]
	Plasma-Thermal ALE	$O_{2}$	Formic acid	42	[144]
Co	Thermal ALE	${\mathrm{Cl}}_{2}$	hfacH/acac	16	[147]
	Plasma-Thermal ALE	$O_{2}$	Formic acid	28	[148]
	Plasma ALE	${\mathrm{Cl}}_{2}$	hfacH	2	[147]
W	Plasma ALE	$O_{2}$	${\mathrm{WF}}_{6}$	6.3	[149]
	Plasma ALE	$O_{2}$/$O_{3}$	${\mathrm{BCl}}_{3}\to$ HF	2.5	[114]
	Thermal ALE	${\mathrm{NF}}_{3}$	$O_{2}$	2.6	[150]
	Thermal ALE	${\mathrm{Cl}}_{2}$	Ar	2.1	[116]
Cu	Plasma-Thermal ALE	$O_{2}$	Formic acid	37	[144]
	Thermal ALE	$O_{2}$/$O_{3}$	hfac	1	[151]
	Thermal ALE	Acetic acid	$O_{2}$-GCIB	0.7	[152]
Ru	Thermal ALE	$O_{2}$	Ethanol		[145]
	Plasma ALE	30 s HCOOH (0.50 Torr)	2.5 min Ar	0.85 ± 0.15	[153]
Ru	Thermal ALE	$O_{2}$	HCOOH	20 nm/min	[154]
	Thermal ALE	$O_{2}$	hfacH		[155]

**Table 6:** Others.

Materials	Etch Type	Surface Adsorption	Surface Removal	EPC (Å/cycle)	Reference
**Alloy etching**					
Cu alloy	Plasma-Thermal ALE	$O_{2}\left(0\;V,\;500\;W,\;1\;\min\right)$	Formic acid vapor (50 s)	37	[148]
Pt alloy	Plasma-Thermal ALE	$O_{2}\left(0\;V,\;500\;W,\;2\;\min\right)$	Formic acid vapor (50 s)	5	[148]
Pd alloy	Plasma-Thermal ALE	$O_{2}\left(0\;V,\;500\;W,\;3\;\min\right)$	Formic acid vapor (50 s)	12	[148]
Co alloy	Plasma-Thermal ALE	$O_{2}\left(0\;V,\;500\;W,\;4\;\min\right)$	Formic acid vapor (50 s)	28	[148]
Fe alloy	Plasma-Thermal ALE	$O_{2}\left(0\;V,\;500\;W,\;5\;\min\right)$	Formic acid vapor (50 s)	42	[148]
CoFeB	Plasma ALE	${\mathrm{Cl}}_{2}\left(500\;W,50\;W\right)$	$H_{2}\left(800\;W,\;50\;W\right)$		[144]
	Thermal ALE	$O_{2}$	Formic acid	18	[163]
	Thermal ALE	chlorine	acacH	1.5	[164]
SiGe	Thermal ALE	O${}_{2}$	${\mathrm{CF}}_{4}/C_{4}F_{8}\left(\mathrm{Alternately}\right)$	2.3	[159]
	Thermal ALE	O${}_{2}$/$O_{3}$	HF TMA	0.57/0.42	[166]
**Two dimensional material etching**					
Graphene	Plasma ALE	$O^{2+}/O^{+}\left(100\;\mathrm{sccm}\right)$	${\mathrm{Ar}}^{+}\left(11.2\;\mathrm{eV}\right)$	7.3	[165]
MoS${}_{2}$	Thermal ALE	Cl radical (produced by ICP)	Ar${}^{+}$		[165]
	Plasma ALE	${\mathrm{SF}}_{6}\;+\;N_{2}$ plasma		2.8–3.6 nm/min	[167]
MoSe${}_{2}$	Plasma ALE	${\mathrm{SF}}_{6}\;+\;N_{2}$ plasma		0.18 Å/s	[168]

## Data Availability

Not applicable.
